# High‐Efficiency Solution‐Processable OLEDs by Employing Thermally Activated Delayed Fluorescence Emitters with Multiple Conversion Channels of Triplet Excitons

**DOI:** 10.1002/advs.202101326

**Published:** 2021-07-26

**Authors:** Yuchao Liu, Lei Hua, Zhennan Zhao, Shian Ying, Zhongjie Ren, Shouke Yan

**Affiliations:** ^1^ State Key Laboratory of Chemical Resource Engineering College of Materials Science and Engineering Beijing University of Chemical Technology Beijing 100029 China; ^2^ Key Laboratory of Rubber‐Plastics Ministry of Education Qingdao University of Science & Technology Qingdao 266042 P.R. China

**Keywords:** low efficiency roll‐off, multiple conversion channels, organic light emitting diodes (OLEDS), thermally activated delayed fluorescence (TADF), triplet excitons

## Abstract

The state‐of‐the‐art luminescent materials are gained widely by utilizing thermally activated delayed fluorescence (TADF) mechanism. However, the feasible molecular designing strategy of fully exploiting triplet excitons to enhance TADF properties is still in demand. Herein, TADF emitters with multiple conversion channels of triplet excitons are designed by concisely halogenating the electron acceptors containing carbonyl moiety. Compared with the chlorinated and brominated analogues, the fluorinated emitter exhibits distinguishing molecular stacking structures, participating in the formation of trimers through integrating C—H···F and C═O···H hydrogen bonds together. It is also demonstrated that the multiple channels can be involved synergistically to accelerate the spin‐flip of triplet excitons, and to take charge of the relatively superior reverse intersystem crossing constant rate of 6.20 × 10^5^ s^–1^, and thus excellent photoluminescence quantum yields over 90% can easily be achieved. Then the solution‐processable organic light emitting diode based on fluorinated emitter can achieve a record‐high external quantum efficiency value of 27.13% and relatively low efficiency roll‐off with remaining 24.74% at 1000 cd m^−2^. This result manifests the significance of enhancing photophysical properties through constructing multiple conversion channels of triplets excitons for high‐efficiency TADF emitters and provides a guideline for the future study.

## Graphical abstract

The multiple conversion channels of triplet excitons are involved in fluorinated thermally activated delayed fluorescence emitters to accelerate the spin‐flip of triplet excitons and reverse intersystem crossing process. Eventually, the photoluminescence quantum yields can surpass over 90%, and the solution‐processable organic light emitting diodes can achieve a record‐high external quantum efficiency of 27.13% and relatively low efficiency roll‐off.

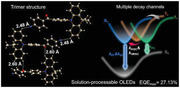

## 1 Introduction

Since the thermally activated delayed fluorescence (TADF) mechanism was incorporated into organic light emitting diodes (OLEDs) field, a growing number of TADF emitters covering the luminous colors from deep blue to near infrared have successfully been designed and synthesized in succession.^[^ ^1^, ^2^, ^3^ ^]^ For TADF emitters, the most widely recognized mechanism is to harness the singlet and triplet excitons simultaneously through endothermic reverse intersystem crossing (RISC) process. In this way, 100% internal quantum efficiency can be realized theoretically, as the result of small singlet–triplet energy level splitting (*ΔE*_ST_). Generally, the small *ΔE*_ST_ values can be obtained by limited overlap integral between highest occupied molecular orbital (HOMO) and lowest unoccupied molecular orbital (LUMO), which can be induced by twisted donor‐acceptor (D–A) moieties. Materials chemistry has been allowed to access the high efficiency TADF emitters. In practice, the effective chemical modification with precise control over donor/acceptor moieties,^[^ ^4^, ^5^, ^6^ ^]^ joint mode between donors and acceptors,^[^ ^7^, ^8^ ^]^ substituent groups^[^ ^9^, ^10^, ^11^ ^]^ from a typical D‐Askeleton have been adopted. As a result, the external quantum efficiency (EQE) values have achieved over 35% for blue^[^ ^12^ ^]^ and green^[^ ^13^ ^]^ OLEDs, and nearly 30% for red^[^ ^14^, ^15^ ^]^ OLEDs fabricated by vacuum deposition technology.

Besides materials chemistry, the intermolecular packing modes or molecular ordering in the solid states also play a crucial role in the photophysical properties of TADF emitters.^[^ ^16^, ^17^, ^18^ ^]^ For example, Xu and co‐workers recently proposed the rigidly fixed configuration of highly distorted TADF emitter with multiple donor moieties via intra‐ and inter‐molecular C—H···F interactions, which improves the TADF properties dramatically.^[^ ^16^ ^]^ The favorable molecular configurations and arrangements for efficient TADF can be constructed through typical intermolecular interactions, such as, hydrogen bond, *π*–*π* stacking, C—H···*π*, and C═O···*π*.^[^ ^17^, ^19^, ^20^, ^21^ ^]^ As a consequence, the triplet‐to‐singlet conversion and radiation decay are boosted due to the reduced energy barrier and optimized transition states.^[^ ^22^, ^23^, ^24^ ^]^ Therefore, it is necessary and reasonable to control the stacking modes of TADF emitters to optimize the emission performance.

**Figure 1:**
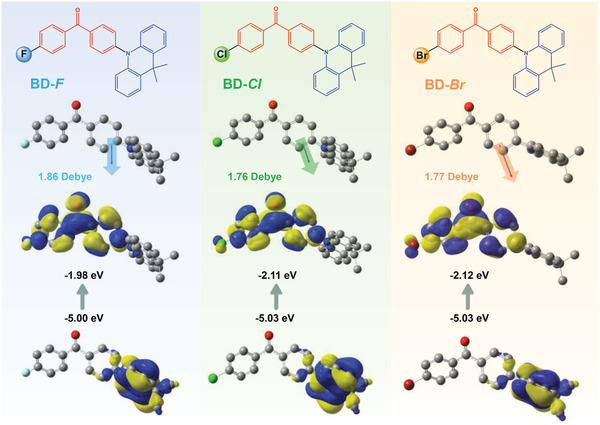
The molecular structures of halogenated emitters and the optimized structures performed by Gaussian 09 software package using CAM‐B3LYP/6‐31G (d, p) level. Molecular dipole moments in the optimized molecular models for emitters, frontier molecular orbital distributions, and the corresponding energy levels are also shown in the diagrams.

Nonetheless, the mechanisms and approaches of regulating the molecular packing modes via intermolecular interactions are still unsatisfactory, especially for succinct D—A type TADF molecules. Moreover, the effect of molecular configurations on transition processes and excited‐state nature of TADF emitters are also far from being well understood.^[^ ^16^, ^17^, ^25^, ^26^ ^]^ In this context, to establish multiple valid intermolecular interactions, carbonyl groups and halogen atoms were introduced into our prototype TADF emitters. That is, carbonyl‐containing benzophenone is used as the electron acceptor and then the substituting halogen atoms including fluorine, chlorine, and bromine are incorporated at para position of carbonyl group as shown in **Figure** **1**. In this way, the different kinds of intermolecular weak interactions with various strengths can be constructed to figure out the relationship between molecular packing modes and final photophysics. Herein, TADF molecules of 4‐(9,9‐dimethyl‐9,10‐dihydroacridine) benzophenone as skeleton integrated with fluorine (BD‐F), chlorine (BD‐Cl), and bromine (BD‐Br) substituents, were prepared, respectively. In the single crystal of these TADF molecules, dimers can form through C═O···H hydrogen bonds for three emitters, and even trimers can form for BD‐F through integrating C—H···F and C═O···H hydrogen bonds together. These favorably constructed structures can rigidify the molecular conformations of the excited‐state in some excitons, and thus suppress the nonradiative pathways of excitons. Additionally, we also demonstrated that the multiple conversion channels of triplet excitons could be involved synergistically to accelerate the spin‐flip of triplet excitons, and consequently to take charge of the superior RISC dynamics, and thus the superior parameter of photoluminescence quantum yield (PLQY) over 90% could be reached, especially for BD‐F based blended films. Finally, the solution‐processable OLEDs devices based on BD‐*F* can achieve a record‐high EQE value of 27.13% and relatively low efficiency roll‐off with remaining 24.74% at 1000 cd m^−2^. Our strategy accomplishes to boost the luminescence efficiency of OLEDs through constructing multiple conversion channels of triplet excitons.^[^ ^27^, ^28^ ^]^

## 2 Results and Discussion

The detailed synthetic routes of TADF emitters are shown in Schemes S1–S3, Supporting Information. The chemical structures of the resultant emitters were characterized by ^1^H NMR, ^13^C NMR, mass spectrometry, and elemental analyses (Figures S1–S4 and Table S1, Supporting Information). The halogenated molecules were first investigated by quantum‐chemical calculations. As shown in Figure 1, the donor and acceptor subunits of optimized ground‐state molecules exhibit nearly orthogonal geometries. Moreover, the HOMO and LUMO are found to be located on the donor and acceptor moieties, respectively. The molecular dipole moment (µs_0_) of three emitters is 1.86 Debye of BD‐F, 1.76 Debye of BD‐Cl, and 1.77 Debye of BD‐Br, respectively. Moreover, these emitters have almost similar HOMO levels, whereas the LUMO levels gradually decline with the increasing atomic numbers of halogens. According to excited‐state analysis using time‐dependent density functional theory (TD‐DFT), the transition dipole moment (*Δ*µs_1–s0_) changes nearly in an opposite trend with µs_0_ values, enhancing from 27.54 Debye of BD‐F to 28.57 Debye of BD‐Cl and 28.62 Debye of BD‐Br (Figure S5, Supporting Information). It should be noted that the µs_0_ and *Δ*µs_1–s0_ values for BD‐Cl are quite close to those for BD‐Br while different from BD‐F, indicating that BD‐Cl and BD‐Br are likely to perform different photophysical properties with BD‐F.

To gain further insight into the excited‐state characteristics, we performed natural transition orbital analysis of lowest singlet (S_1_) and triplet excited states (T_1_). As shown in Figure S6, Supporting Information, the hole and particle distributions are totally separated with tiny overlaps at boundary, thus indicating definite intramolecular charge transfer natures of S_1_ and T_1_ states for these emitters.^[^ ^29^, ^30^ ^]^ Furthermore, we also carried out spin‐orbit coupling matrix element (SOCME) calculations between S_1_ and T_1_ states using ORCA 4.1.1 package at CAM‐B3LYP/G 6–31g(d, p), and as a result, the SOCME values for these emitters can essentially be neglected in their single molecule states (Table S2, Supporting Information).^[^ ^10^, ^18^ ^]^

**Figure 2:**
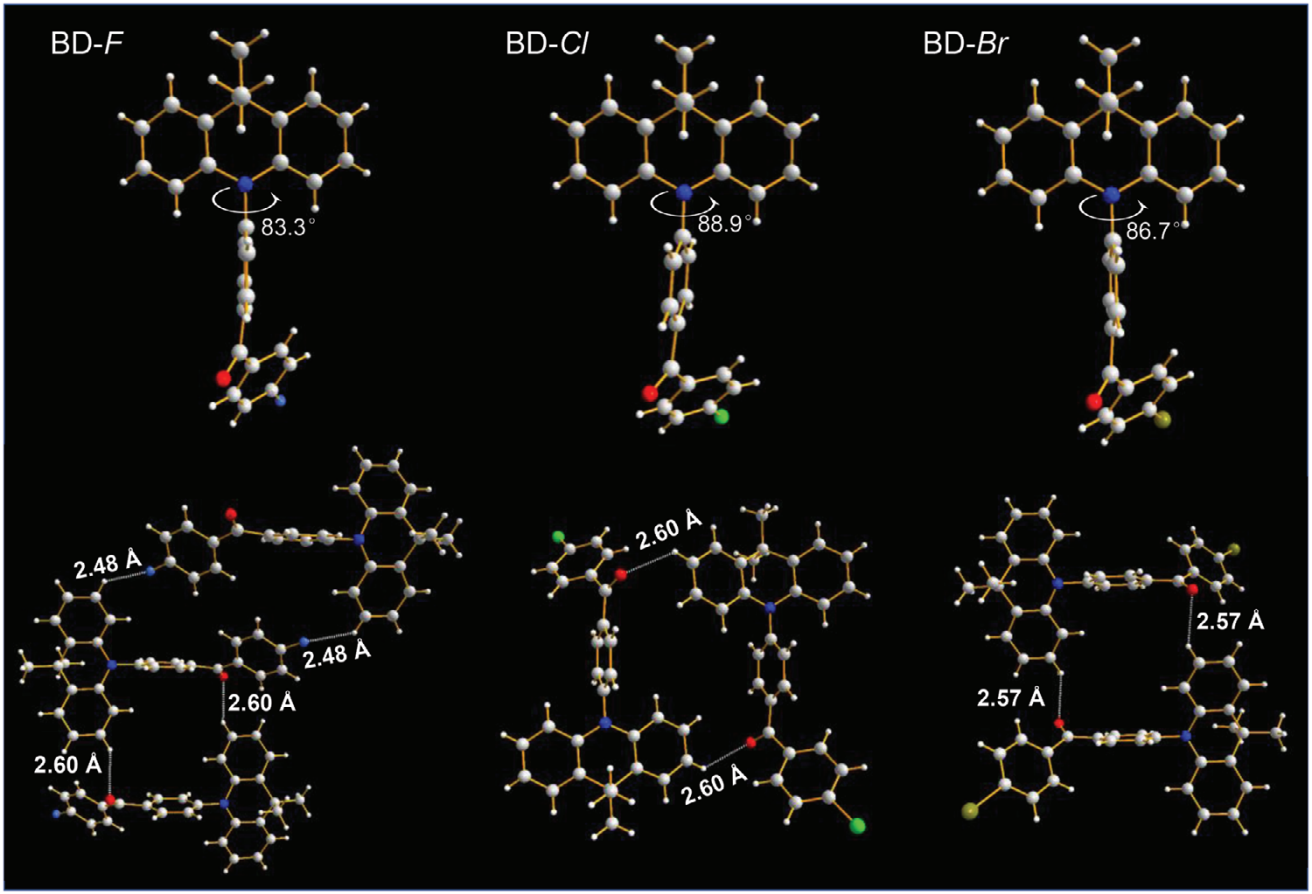
The single crystal structures of the halogenated emitters and the corresponding stacking structures, neglecting the interaction with the distance over 3.5 Å.

In **Figure** **2**, the single crystal structures of these halogenated emitters are quite similar to the simulation results, featuring a nearly orthogonal dihedral angle between the donor and acceptor moieties. The dihedral angle exhibits minor variation with changing halogen atoms, from 83.3° of BD‐F to 86.7° of BD‐Br and 88.9° of BD‐Cl. Remarkably, the intermolecular assembly can indeed be constructed through bimolecular or trimolecular interactions, highlighting that the halogen modifying strategy enables regulation of packing modes for D—A type TADF molecules. As a result, the molecular rotations and vibrations, and the unfavorable intermolecular interactions might be partly suppressed due to the rigid and restricted dimer or trimer structures. In this case, the excited‐state configuration would be fixed, and nonradiative transition can also be restrained.^[^ ^16^, ^31^ ^]^ Actually, two kinds of intermolecular assemblies form through intermolecular hydrogen bonds as desired. Concretely, the central BD‐F molecule participates in the formation of trimer through integrating C—H···F and C═O···H hydrogen bonds with the surrounded neighboring molecules,^[^ ^16^, ^32^, ^33^ ^]^ and the bond lengths are 2.48 Å and 2.60 Å. Whereas BD‐Cl and BD‐Br molecules form the similar dimers through only C═O···H hydrogen bonds, and the bond lengths are 2.60 Å and 2.57 Å, respectively. The individual trimer construction of BD‐F can enable more delocalized distribution of excited states, and thus the excited‐state nature and the transient decay performance might be further optimized to promote the upconversion of triplet excitons.^[^ ^34^ ^]^

**Figure 3:**
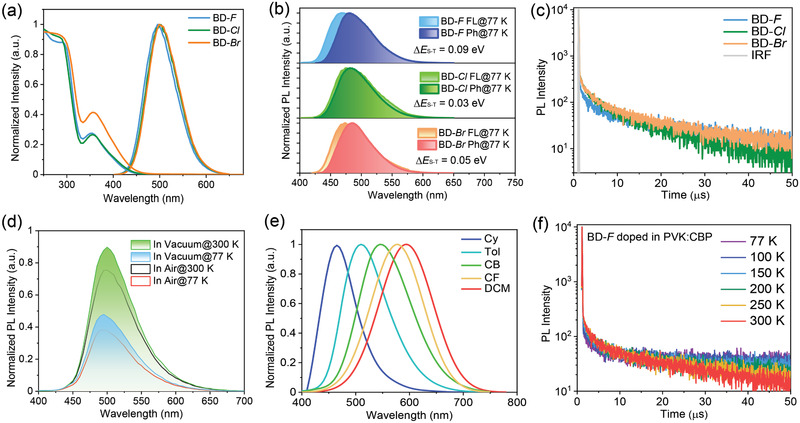
a) The ultraviolet–visible absorption spectra and PL spectra of 20 wt% emitters blended with PMMA matrix. b) The fluorescence and phosphorescence spectra of emitters detected in toluene at 77 K. c) The transient PL spectra of 20 wt% emitters blended with 20 wt% PVK and 60 wt% CBP in vacuum condition. d) The steady‐state PL spectra of blended films for BD‐F in air and vacuum conditions at room temperature and 77 K. e) Emission spectra of BD‐F diluted in different solvents at room temperature. f) The temperature‐dependent transient PL decay spectra of the blended BD‐F films.

The ultraviolet–visible absorption spectra of emitters experimentally confirm the presence of intense charge transfer (CT) transition band ≈360 nm diluted both in poly(methyl methacrylate) (PMMA) matrix or toluene (**Figure** **3**a; Figure S7, Supporting Information). From the photoluminescence (PL) spectra, the emission peaks of emitters blended into PMMA matrix are mainly located at ≈500 nm, and PL spectra exhibit discernible redshift with increasing the atomic number of halogens, which is in accordance with the improved *Δ*µs_1‐s0_ values mentioned above. As the *ΔE*_ST_ value is a prerequisite for maintaining valid upconversion of triplet excitons, we then detected the energy splitting of these emitters. The energies of S_1_ and T_1_ states can be estimated from the onset values of fluorescence and phosphorescence spectra in toluene at 77 K as shown in Figure 3b. In good consistence with the variation of dihedral angles between the donor and acceptor moieties, the calculated *ΔE*_ST_ values are 0.09, 0.05, and 0.03 eV for BD‐F, BD‐Br, and BD‐Cl, respectively, which can ensure the establishment of effective channels for the triplet‐to‐singlet RISC process. Furthermore, compared with the emissions in dilute solution, the bathochromic shift in the solid films suggests the existence of strong intermolecular interactions as well.

The transient PL spectra demonstrate bi‐exponential decay features of 20 wt% emitters blended with 20 wt% polyvinylcarbazole (PVK, *M*_w_ = 200 000 ) and 60 wt% 4,4′‐bis(N‐carbazolyl)‐1,1′‐biphenyl (CBP) under vacuum condition, including nanosecond‐scale prompt fluorescence (PF) and microsecond‐scale delayed fluorescence (DF) components (Figure 3c). It is noteworthy that the lifetime (*τ*_p_) of PF component declines from 21.0 ns of BD‐F to 19.4 ns of BD‐Cl and 18.7 ns of BD‐Br, respectively, which can be ascribed to the increased *Δ*µs_1‐s0_ values and the corresponding high oscillator strengths. Surprisingly, BD‐F exhibits unpredictably shorter DF lifetime (*τ*_d_) of 7.8 µs than BD‐Cl of 11.8 µs and BD‐Br of 11.3 µs, although the BD‐Cl and BD‐Br might possess potential heavy atom effect, which illustrates that the photophysical properties can be enhanced by fully exploiting triplet excitons for BD‐F.

To evaluate the extent of triplet exciton participating in luminescence process, we gained the DF values through integrating the steady‐state PL spectra in air (*S*_air_) or vacuum (*S*_vac_) (Figure 3d; Figure S8, Supporting Information). The calculated DF components ( *φ*_DF_ = *S*_vac_/(*S*_vac_ + *S*_air_) ) are possibly related to the permeability of oxygen into the films of TADF materials. The *φ*_DF_ values of BD‐F, BD‐Cl, and BD‐Br at room temperature are 13.3%, 11.7%, and 10.8%, respectively, while these values are boosted to 18.9%, 15.3%, and 13.8% at 77 K, demonstrating that confined configuration of triplet excited‐state at low temperature can enhance PL performance despite the suppressed RISC process. Additionally, the blueshift of PL spectra measured at low temperature should be attributed to the attenuated molecular vibrations according to Frank–Condon theory. Then PLQY values of the blended films were directly measured in air as shown in Figure S9, Supporting Information. We also optimized the doping ratio of emitters and two hosts and even changed the host materials (Table S4, Supporting Information), but eventually the blended films consisting of 20 wt% emitters, 20 wt% PVK, and 60 wt% CBP displayed the highest PLQY here. After integrating with *φ*_DF_ values of these emitters, the PLQY values are 95.3% for BD‐F, 85.4% for BD‐Cl, and 88.2% for BD‐Br, respectively (**Table** **1**). The extremely high PLQY values even in air should be ascribed not only to the rapid spin‐flip of triplet excitons, but also to the encapsulation of host matrix and the suppressed nonradiative decay induced by special packing modes.

**Table 1:** Summary of photophysical properties of the halogenated emitters

Emitter	*λ*_em_ [nm]	*ΔE*_ST_ [eV]^c)^	*τ*_p_ [ns]^d)^	*τ*_d_ [µs]^e)^	*φ*_DF_ [%]^f)^	*Φ*_PL_ [%]^g)^	*k*_RISC_ [10^5^ s^–1^]^h)^	$k_{\mathrm{nr}}^{T}$
BD‐F	496^a)^/468^b)^	0.09	21.0	7.8	13.3	95.3 ± 0.2	6.20	2.52
BD‐Cl	500/475	0.03	19.4	11.8	11.7	85.4 ± 0.1	2.23	2.75
BD‐Br	500/474	0.05	18.7	11.3	10.8	88.2 ± 0.2	2.67	2.58
BD‐H	498/472	0.07	21.4	12.2	9.6	73.9 ± 0.2	1.63	3.04

^a)^ The peak values of PL spectra in PMMA;

^b)^ The peak values of PL spectra in toluene at 77 K;

^c)^ The energy splitting between S_1_ and T_1_ states obtained from the onset values of fluorescence and phosphorescence spectra in toluene at 77 K;

^d)^ The lifetime of prompt fluorescence component;

^e)^ The lifetime of delayed fluorescence component;

^f)^ The integrated ratio of delayed fluorescence (DF) component through integrating the steady‐state PL spectra under air (*S*_air_) or vacuum condition (*S*_vac_), *ϕ*_DF_ = *S*_vac_/(*S*_vac_ + *S*_air_);

^g)^ The photoluminescence quantum yields integrated by the *φ*_DF_ values and the quantum yield of blended films measured in air condition;

^h)^ The rate constant of reverse intersystem crossing calculated from $k_{\mathrm{RISC}}=\frac{\emptyset_{\mathrm{PL}}}{\tau_{d}\times (1-\emptyset_{\mathrm{DF})}}\;$, where the *Φ*_P_ is the ratio of prompt fluorescence component, and the ∅_DF_ values here are determined by fitting transition decay curves, 80.3% for BD‐F, 67.5% for BD‐Cl, 70.8% for BD‐Br, and 62.9% for BD‐H;

^i)^ The nonradiative decay rate of triplet exciton calculated from $k_{\mathrm{nr}}^{T}=\frac{1-\emptyset_{\mathrm{DF}}}{\tau_{d}}$.

To characterize the spin‐flip processes quantificationally, we summarized the rate constants of RISC (*k*_RISC_) and nonradiative decay ($k_{\mathrm{nr}}^{T}$) in Table 1. The *k*_RISC_ value of BD‐F is 6.20 × 10^5^ s^–1^, exceeding 2.23 × 10^5^ s^–1^ of BD‐Cl and 2.67 × 10^5^ s^–1^ of BD‐Br, despite the similar $k_{\mathrm{nr}}^{T}$ values of 2.52 × 10^4^ s^–1^ for BD‐F, 2.75 × 10^4^ s^–1^ for BD‐Cl, and 2.58 × 10^4^ s^–1^ for BD‐Br, suggesting the existence of underlying decay mechanism for BD‐F, which will be discussed later. Therefore, it is rational that the kinetic process of radiative transition can be regulated by our designing strategy. Additionally, we also noticed that BD‐Br exhibits slightly higher *k*_RISC_ value than BD‐Cl, in accordance with the tendency of their PLQY values. It can be ascribed to the enhanced spin orbit coupling (SOC) interaction in the presence of heavy atoms.^[^ ^35^, ^36^, ^37^ ^]^ Compared with the chlorinated compound, the brominated one can possibly increase SOC effect due to the higher atomic number. In this way, the enhanced *k*_RISC_ value and the corresponding higher PLQY value can be observed in BD‐Br because of accelerated conversions of triplet excitons between S_1_ and T_1_ states induced by more intense SOC effect. To verify the heavy atoms effect presented in chlorinated and brominated compounds, we also investigated the photophysical properties of non‐halogenated analogue, BD‐H. As shown in Figure S10, Supporting Information; Table 1, the *τ*_p_ and *τ*_d_ values of BD‐H are 21.4 ns and 12.2 µs, which are relatively higher than those of halogenated emitters, and as a result, the *k*_RISC_ value of BD‐H is 1.63 × 10^5^ s^–1^, which is correspondingly lower due to the absence of heavy atoms effect. It's perhaps important to point out that although the calculated SOCME values of these emitters can be neglected in single molecule state, the SOC effect can still play a critical role for speeding up the conversion of excitons in packing states with much more delocalized distribution of excited state and more components of locally excited states.

With increasing the solvent polarity, the emission spectrum of BD‐F distinctly broadens and redshifts from 470 nm in cyclohexane to 510 nm in toluene, 545 nm in chlorobenzene, 576 nm in chloroform, and 594 nm in dichloromethane (Figure 3e), and the Lippert–Mataga fitting plot (*ν*_abs_–*ν*_em_ against polarizability of solvent) exhibits a slope of over 16 000 cm^–1^ (Figure S11, Supporting Information), well consistent with the strong CT character of S_1_ state mentioned previously. Comparatively, the solvatochromic shifts for BD‐Cl and BD‐Br are more noticeable with slope of ≈19 000 cm^–1^ (Figure S11, Supporting Information), in accordance with the calculated transition dipole moments. Additionally, temperature‐dependent time‐resolved PL spectra of the blended films were recorded to verify the endothermic RISC process (Figure 3f; Figure S12, Supporting Information). As expected for typical TADF characteristics, both the proportion and the lifetime of DF component obviously increase with temperature for all the emitters.

**Figure 4:**
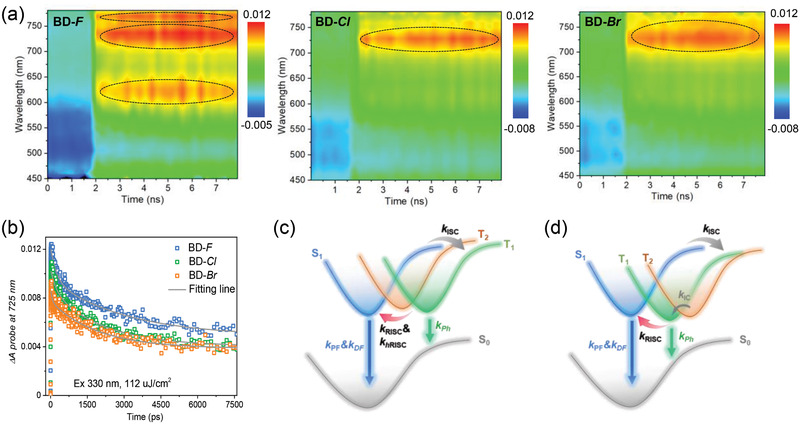
a) The transient absorption (TAS) spectroscopy for the emitters diluted in PMMA matrix. b) The kinetic decay processes of triplet excitons for all emitters detected at 725 nm. c) The schematic diagram of multiple radiative decay channels for BD‐F emitter. d) The schematic diagram of single radiative decay channels for conventional TADF emitters. *k*_PF_ and *k*_DF_ are the decay rate constants of prompt and decayed fluorescence, respectively, and the *k*_Ph_ is the decay rate constant of phosphorescence. *k*_RISC_ and *k*_hRISC_ are the rate constants of RISC of triplet exciton from T_1_ to S_1_ and the RISC from high‐laying triplet states to S_1_, respectively. *k*_ISC_ is the intersystem crossing rate constant from singlet to triplet states, and *k*_IC_ is intrasystem crossing rate constant from high‐laying to low‐laying states.

To gain further insight into the synergistic effect presented in these emitters and confirm experimentally the presence of multiple conversion channels of triplet excitons especially in BD‐F, we performed pump‐probe transient absorption (TAS) spectroscopy for the emitters diluted in PMMA matrix (**Figure** **4**a). The pump wavelength is 330 nm, and detected range is 400–780 nm. All the measurements were carried out at room temperature. From the TAS contour maps, all these emitters exhibit broad photoinduced absorption (PIA) signals at ≈725 nm after 2000 ps, assigning to the triplet‐state feature. Coupled with the preliminary theoretical calculation results of dominant CT natures for S_1_ and T_1_ states, we can infer that this PIA signal is the absorption band of charge–transfer triplet excited state (^3^CT), and the decay process mainly corresponds to the hyperfine coupling (HFC) interaction between ^3^CT and charge–transfer singlet excited state. The kinetic decay processes of triplet excitons for these emitters detected at 725 nm were also studied as shown in Figure 4b, and the fitted decay lifetimes of triplet exciton were summarized in Table S3, Supporting Information. In consequence, the lifetimes gradually reduce along with the enlarged atomic numbers, which is well consistent with the *τ*_d_ of BD‐Cl and BD‐Br detected from transient PL decay spectra.

Intriguingly, the TAS contour map of BD‐F also comprises two extra PIA signals at ≈625 and 760 nm, nevertheless not observed in other emitters. The PIA signal at ≈625 nm can be assigned to the higher‐lying excited triplet state (T_2_), while the PIA signal at ≈760 nm, just slightly lower than ^3^CT, is the locally excited triplet state, which is generally recognized as intermediate state in the SOC interaction between S_1_ and T_1_.^[^ ^23^, ^38^, ^39^ ^]^ This result implies that the promising multiple conversion channels for triplet exciton are established in BD‐F films, including the coexisting of HFC and SOC interactions between S_1_ and T_1_, and the rapid RISC from high‐lying triplet state of T_2_ to S_1_ (Figure 4c).^[^ ^40^ ^]^ Oppositely, there is only one available pathway toward emitting state from excited triplet state for BD‐Cl and BD‐Br emitters (Figure 4d). In addition, the energy gap between T_1_ and T_2_ is ≈0.35 eV from the TAS results of PIA signals at ≈625 and 760 nm for BD‐F, meanwhile, this value is 0.37 eV determined from excited‐state DFT calculation, indicating that the TAS analysis in this work is reasonable and credible to study excited‐state nature of TADF molecules. Eventually, we can conclude that, in the case of BD‐F emitter, the multiple conversion channels of triplet excitons are engaged synergistically to accelerate the spin‐flip of triplet excitons, and consequently to take charge of the unexpectedly excellent RISC dynamics.^[^ ^22^, ^41^, ^42^, ^43^ ^]^

**Figure 5:**
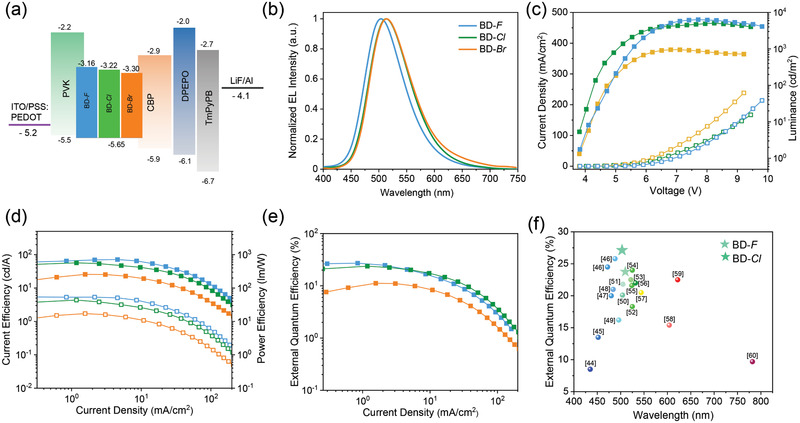
a) The solution‐processable device architecture and the energy levels. b) The electroluminescence spectra detected at 7 V. c) Current density–voltage‐luminance curves. d) The curves of power efficiency and current efficiency versus current density. e) The curves of EQE value versus current density. f) The reported EQE values of solution‐processed devices employing TADF small molecules as emitters.

To verify the potential of these emitters in electroluminance (EL) devices, we fabricated solution‐processable OLEDs with the architecture of ITO/PSS: PEDOT 4083 (40 nm)/emitter (40 nm)/DPEPO (7.5 nm)/TmPyPB (45 nm)/LiF (0.5 nm)/Al (110 nm) shown in **Figure** **5**a, where PSS: PEDOT, DPEPO, and TmPyPB are poly (3,4‐ethylenedioxythiophene)‐poly(styrene sulfonate), bis[2‐(diphenylphosphino) phenyl]ether oxide, and 1,3,5‐tri(m‐pyridin‐3‐ylphenyl)benzene, respectively. The PSS: PEDOT 4083 was adopted as hole injecting and transferring layer, and DPEPO and TmPyPB served as exciton‐blocking and electron‐transferring layer. For the emitting layer, the halogenated emitters were embedded into mixed host of PVK and CBP, which could not only enhance the charge transferring capability and restrain the aggregation induced concentration quenching of excitons, but also improve the film‐forming ability and morphology of emitting layer. The morphology of the emitting layer has also been revealed by atomic force microscopy as shown in Figure S13, Supporting Information. The values of root mean‐square surface roughness for three emitters are all ≈0.25 nm, indicating excellent film‐forming properties. The energy levels of emitters were determined by cyclic voltammetry measurement (Figure S14, Supporting Information) to guarantee good matching with the energy levels of transferring layers.

Similar with the PL spectra, EL peaks are located at ≈500 nm, exhibiting slight redshift with the enlarged atomic number of halogens (Figure 5b). From the current density–voltage–luminance curves shown in Figure 5c; Figure S15, Supporting Information, the turn‐on voltages (*V*_on_) of devices are comparable to each other at ≈3.6–3.8 V. The luminance can exceed 5000 cd m^−2^ at 8 V for BD‐F and BD‐Cl based devices (20 wt% emitters: 20 wt% PVK: 60 wt% CBP), while the maximum luminance (*L*_max_) is compromised for BD‐Br below 1000 cd m^−2^. The maximum current (*CE*_max_) and power (*PE*_max_) efficiencies are 71.4 cd A^−1^ and 54.2 lm W^−1^ for BD‐F, while it is 57.0 cd A^−1^ and 43.7 lm W^−1^ for BD‐Cl, which are also much higher than those for BD‐Br of 25.8 cd A^−1^ and 17.2 lm W^−1^ (Figure 5d). An important observation is that the device performances based on BD‐F and BD‐Cl matches well with their corresponding photophysical properties. However, the devices based on BD‐Br exhibit unexpected inferior performance, even though BD‐Br exhibits relatively good photophysical properties as well. With careful consideration, we inferred that the inconsistent relationship between photophysical properties and device performance of BD‐Br should be blamed on its low thermal stability. The low thermal decomposition temperature of BD‐Br ≈200 °C is far lower than BD‐F and BD‐Cl (over 300 °C) (Figure S17, Supporting Information). Thus, BD‐Br molecules would disintegrate under thermal treatment or heat evaporation process for fabricating devices. Finally, the generated free radicals will dramatically quench the emitters as exciton quenchers, and result in poor luminescence properties and device stability. Additionally, for BD‐H based device, the *CE*_max_ and *PE*_max_ are 51.4 cd A^−1^ and 33.6 lm W^−1^, respectively, which is lower than that of BD‐Cl with the same device architecture (Figure S16, Supporting Information; **Table** **2**). Therefore, the device performance can be demonstrated to be consistent well with the photophysical properties for halogenated and non‐halogenated analogues, and the the heavy atoms effect presented in halogenated compounds, especially for BD‐Cl can be further confirmed.

**Table 2:** EL properties of solution‐processed devices employing the halogenated emitters

Emitter	*λ*_EL_ [nm]^a)^	*V*_on_ [V]^b)^	*L*_max_ [cd m^−2^]^c)^	*CE*_max_ [cd A^−1^]^d)^	*PE*_max_ [lm W^−1^]^e)^	*EQE*_max_ [%]^f)^	*EQE*_1000_ [%]^g)^	CIE^h)^
BD‐F	503	3.6	6350	71.4	54.2	27.13	24.74	(0.236, 0.494)
BD‐Cl	512	3.7	5081	57.0	43.7	23.76	20.20	(0.263, 0.530)
BD‐Br	513	3.8	951	25.8	17.2	11.19	—	(0.272, 0.527)
BD‐*H*	506	4.0	2816	51.4	33.6	20.60	13.40	(0.245, 0.506)

^a)^ The peak value of electroluminescence;

^b)^ Turn‐on voltage at 1 cd m^−2^;

^c)^ Maximum luminance;

^d)^ Maximum current efficiency;

^e)^ Maximum power efficiency;

^f)^ Maximum external quantum efficiency;

^g)^ Maximum external quantum efficiency at 1000 cd m^−2^;

^h)^ Coordinates of Commission Internationale de L'Eclairage.

Accordingly, the values of*EQE*_max_ can reach 27.13% and 23.76% for BD‐F and BD‐Cl based devices, respectively, which are much higher than BD‐H based device of 20.60%, BD‐Br based device of 11.19% (Figure 5e; Figure S15, Supporting Information). It is especially noteworthy that BD‐F and BD‐Cl based devices exhibit relatively low efficiency roll‐off with remaining 24.74% and 20.20% at 1000 cd m^−2^, respectively, corresponding to 8.8% and 14.8% decrease in EQE. The dramatically suppressed efficiency roll‐off for BD‐F can be assigned to the fast *k*_RISC_ from the multiple conversion channels of triplet excitons and the reduced nonradiative decay. All these EL properties are summarized in Table 2; Table S4, Supporting Information. As shown in Figure S15 and Table S4, Supporting Information, we have also fabricated OLEDs using other hosts, such as PVK & 1,3‐bis[2‐(4‐tert‐butylphenyl)‐1,3,4‐oxadiazo‐5‐yl]benzene (OXD‐7) (device D) and PVK & 9‐(3‐(9H‐Carbazol‐9‐yl)phenyl)‐9H‐carbazole‐3‐carbonitrile (mCPCN) (device E). The device E also exhibits ≈22% EQE value, indicating the superior performance of BD‐F. Whereas the device D exhibits relatively low EQE value of 18.5%, which might be caused by imbalanced charge injecting and transporting properties when incorporating OXD‐7 or mCPCN into emitting layer as host material. As illustrated in Figurer 5f, to our best knowledge, the EQE value of 27.13% for BD‐F based device is at a record high for solution‐processed OLEDs devices.^[^ ^44^, ^45^, ^46^, ^47^, ^48^, ^49^, ^50^, ^51^, ^52^, ^53^, ^54^, ^55^, ^56^, ^57^, ^58^, ^59^, ^60^ ^]^ It also should be noted that the solution‐processable emitters with deep‐blue or near‐infrared color still obviously lag behind others, and thus we need to pay more concentration on promoting their luminescence efficiency through elaborate molecular designing and structural regulation depicted in our work.

## 3 Conclusion

In summary, we constructed molecular stacking modes of TADF emitters by concisely halogenating D–A type molecules. From the structures of single crystals, it is confirmed that the dimer, even trimer for BD–F, structures can be constructed through different intermolecular hydrogen bonds, highlighting that the halogen modification strategy enables regulation of packing modes for linear D—A type TADF molecules, which can fix the excited‐state configuration, and thus restrain the nonradiative transition. According to the kinetic analysis of transient decay process, it is demonstrated that the radiative transition can be regulated by halogenation, and hence a superior parameter of PLQY over 90% has been achieved. Especially for BD–F, the formation of multiple conversion channels can also synergistically accelerate the spin‐flip of triplet excitons, and accordingly to take charge of the superior RISC dynamics. In accordance with the superior photophysical properties, the solution‐processable OLEDs based on BD‐F can achieve a record‐high EQE value of 27.13% and relatively low efficiency roll‐off with remaining 24.74% at 1000 cd m^−2^, corresponding to an 8.8% decrease in EQE. Consequently, we present a strategy for boosting the luminescence efficiency with precise control over the radiative decay channels via chemical modification.

## 4 Experimental Section

### Device Fabrication and Performance Measurement

Before device fabrication, the ITO glass substrates were precleaned carefully using deionized water, acetone, and isopropanol, successively. After hydrophilic treatment by ultraviolet ozone for 5 min, the PSS: PEDOT aqueous solution was spin‐coated on the ITO substrates. Then, the substrates were transferred into the glovebox and heat‐treated at 120 °C to remove the residual water. The mixture of 2 mg emitting material, 6 mg CBP, and 2 mg PVK was dissolved into 1 mL anhydrous chlorobenzene at 60 °C for 5 h without any stirring. Then the mixture solution was spin‐coated on the PSS: PEDOT films. After heat‐treatment for another 30 min at 60 °C, all the substrates were transferred into deposition system. The devices were fabricated under the pressure of below 10^–5^ Torr. The hole blocking layer DPEPO and electron transferring layer TmPyPB were thermally evaporated in succession at a rate of 1.0 and 2.0 Å s^–1^, respectively. After that, the electron injecting layer LiF was carefully deposited on the organic surface at a rate of 0.1 Å s^–1^, and aluminum electrode was thermally evaporated at a rate of 1.0–3.0 Å s^–1^. The electroluminescence characteristics of the devices were measured using a Keithley 2400 source meter at room temperature. The electroluminescence spectra and luminance of the devices were obtained on a PR670 spectrometer.

## Conflict of Interest

The authors declare no conflict of interest.

## Supporting information

Supporting Information

## Data Availability

Research data are not shared.
