# Supplementary material for: High‐Efficiency Solution‐Processable OLEDs by Employing Thermally Activated Delayed Fluorescence Emitters with Multiple Conversion Channels of Triplet Excitons
Source: Adv Sci (Weinh). 2021 Jul 26;8(18):2101326. doi: 10.1002/advs.202101326 (PMC8456236; doi:10.1002/advs.202101326)
Supplement: Supplementary file 1 — Supporting Information [file ADVS-8-2101326-s001.pdf]

WILEY-VCH

© 2021 Wiley-VCH GmbH

ADVANCED SCIENCE
Open Access

Supporting Information

for *Adv. Sci.,* DOI: 10.1002/advs.202101326

High-Efficiency Solution-Processable OLEDs by Employing Thermally Activated Delayed Fluorescence Emitters with Multiple Conversion Channels of Triplet Excitons

Yuchao Liu[1,2], *Lei Hua*[1], *Zhennan Zhao*[1], *Shian Ying*[2], *Zhongjie Ren*[1,*], *Shouke Yan*[1,2]

S 1. Experimental Procedures

S 1.1 Materials and Characterization

All used reagents were purchased from *J&K Scientific*, *Energy Chemical, Sigma-Aldrich* and other commercial suppliers and used without further purification, unless otherwise noted. Anhydrous dimethyl formamide was purchased from *Aladdin.* Other solvents were purchased from *Beijing Chemical Works*. NMR spectra were performed using a Bruker AVANCE Ⅲ 400 spectrometer (400 MHz). ^{1}H NMR and ^{13}C NMR spectra were obtained in deuterated chloroform as slovent and TMS as internal standard, respectively. Electron spray mass spectra data (ESI-MS) were collected on Xevo G2 Qtof using anhydrous dichloromethane as matrix. Cyclic voltammetry (CV) was performed in nitrogen-bubbled acetonitrile using CHI voltammetric analyser at room temperature. Tetrabutylammonium hexafluorophosphate ($TBAPF_6$ 0.1 M) was adopeted as the electrolyte, and a glassy carbon working electrode, a

platinum wire auxiliary electrode, and an Ag/$AgNO_3$ pseudo-reference electrode were used in the conventional three-electrode system. Cyclic voltammograms were performed from 0 to 1.5 V at scan rate of 100 mV s^{-1}. Then HOMO and LUMO energy levels have been calculated according to the internal reference ferroceneredox couple in acetonitrile by using the following formulas:[1]

$$E_{HOMO} = -\left(E_{(onset,ox\ vs\ Fc^{+}/Fc)} + 4.8\right)$$

$$E_{LUMO} = -\left(E_{(onset,red\ vs\ Fc^{+}/Fc)} + 4.8\right)$$

UV/Vis absorption spectra were recorded on a Hitachi U-2910 spectrophotometer. The PL spectra, including fluorescence at 77 K, and phorescence at 77 K, were recorded on a Hitachi F-7000 fluorescence spectrophotometer, and the energy gap (ΔE_{ST}) between lowest singlet (S_1) and triplet excited states (T_1) was determined from the difference values of the onset positions of fluorescencent and phorescencent spectra. To gain the proportion of delayed fluorescence (DF), the steady-state PL spectra in vacuum and in air were performed using FLS-980 spectrometer from Edinburgh Instruments Limited with Xe lamp source. Transient-state PL spectra in vacuum and in air, including prompt fluorescence (PF) and delay fluorescence (DF) spectra, and the temperature dependence of transient PL decay curves, were determined using nanosecond gated luminescence and lifetime measurements with a high-energy pulsed Nd:YAG laser emitting at 320 nm to fit the transient decay curves using following equation: $\mathrm{A} + \mathrm{B1exp}\,(-t_1/\tau_1) + \mathrm{B2exp}\,(-t_2/\tau_2)$. PLQY was directly obtained from Hamamatsu Absolute PL quantum yield spectrometer C11347 series in air. The TA spectra were recorded by a commercial TA system equipped with a ns pulse laser as the pump source and a broadband laser-driven light source as the probe source. The detector output was fed to a digital oscilloscope, which acquired the waveform and stored it for eventual data processing. The pump wavelength was 330 nm, and detected range is 400-780 nm All the measurements were carried out at room temperature.

S 1.2 Molecular Simulation and Calculation

The following procedures were performed to optimize the molecular structures, calculate the energy properties and analyze the excited states of polymer representative fragments. Firstly, the molecular structures were optimized in Gaussian 09 by performing a density functional theory (DFT) calculation in the CAM-B3LYP mode with a 6-31G (d, p) basis set in the ground state. Then according to the optimized results, highest occupied molecular orbital (HOMO) and lowest unoccupied molecular orbital (LUMO) levels, and ground-state (S_0) dipole moments can be obtained. After that, the excited states energy levels and the energy properties of these polymers in the excited states were determined using Gaussian 09 with with a 6-31G (d, p) basis in a time dependent (TD) mode. To boost the calculation precision of the singlet and triplet energy levels, the number of calculated states was set to 10. Hence, the difference of dipole moments between S_0 and S_1, and transition dipole moments from S_1 to S_0 can be obtained from electron excitation analysis of TD-DFT calculations.

In order to get better insight of the natures of excited states, natural transition orbital (NTO) analysis was performed using Multiwfn 3.6 based on Gaussian output results.[2] NTO analysis was selected to analyze the singlet and triplet excited states in which the sum of square of excitation coefficients was close to ideal value of 0.5, and the eigenvalues of occupied NTOs and maximal NTO pairs were also shown. The distributions of hole and electron can be found from the NTO results and the dominate nature, charge transfer (CT) or locally excited state (LE), can be found out. In order to quantitatively determine the natures of singlet and triplet excited states, the overlap integral, S_r, was defined as the proportion of LE characters in the excited states by using the following equation: [3-5]

$$S_r(\boldsymbol{r}) = \sqrt{\rho^{hole}(\boldsymbol{r})\rho^{electron}(\boldsymbol{r})}$$

Where the $\rho^{hole}(\boldsymbol{r})$ and $\rho^{electron}(\boldsymbol{r})$ are the distribution of hole and electron, respectively. So with the intention of analyzing the impact of nature flip quantitatively, the SOC matrix

elements for above three fragments were calculated using the ORCA 4.1 package with CAM-B3LYP/G-6-31G (d, p) method, and five states were considered in the analog computations.

S 1.3 Synthesis and Characterization

S 1.3.1 The synthesis route of BD-*F*

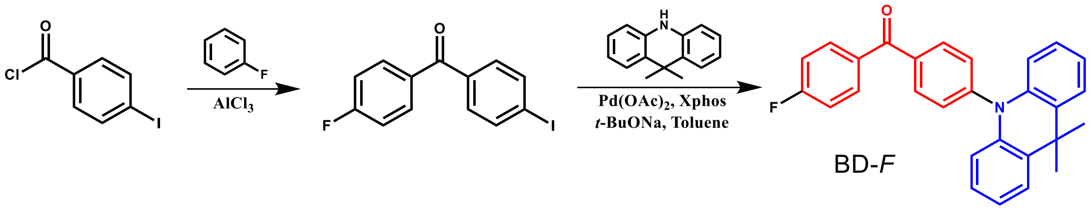

Scheme S1. The synthesis route of BD-*F*.

The compound of **BD-*F*** was obtained following the literature[6].

S 1.3.2 The synthesis route of BD-*Cl*

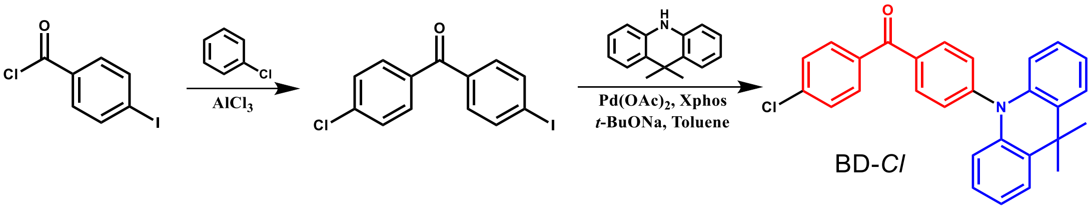

Scheme S2. The synthesis route of BD-*Cl*.

The compound of **BD-*Cl*** was obtained following the literature[6].

S 1.3.3 The synthesis route of BD-*Br*

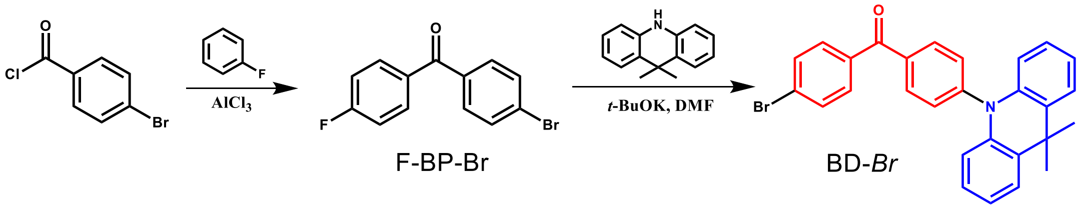

Scheme S3. The synthesis route of BD-*Br*.

a. Synthesis of **F-BP-Br**

4-Bromobenzoyl chloride (2.20 g, 10 mmol) and fluorobenzene (2.40 g, 25 mmol) were added into a 100 ml two-neck flask, and then stirred for 30 min. Then the anhydrous aluminum chloride (2.00 g, 15 mmol) was slowly added into mixture and stirred for another 1 h. After stirring at 60 °C for 6 h, the reaction was cooled down to room temperature. The

reaction was carefully quenched by diluted hydrochloric acid. Until there are no bubbles in the reaction system, abundant dichloromethane was infunded into flask to dissolve the mixture. Then the mixture was washed three times by deionized water and extracted by dichloromethane. After dried by anhydrous magnesium sulfate, the mixture was filtered and concentrated to get crude product. The crude product was further purified by column chromatography on silica gel using petroleum ether/dichloromethane (*v*/*v* = 2/1) as eluent to obtain the title compound of **F-BP-Br** as a pale yellow powder (2.37 g, 90% yield).

^{1}H NMR (400 MHz, $CDCl_3$) δ 7.84 (dd, *J* = 8.6, 5.5 Hz, 2H), 7.66 (s, 4H), 7.19 (t, *J* = 8.6 Hz, 2H).

^{13}C NMR (101 MHz, $CDCl_3$) δ 194.13, 166.78, 164.25, 136.22, 133.39, 132.53, 131.38, 127.56, 115.74.

LC-MS m/z [M+H] calculated for C13H8FBrO+H 278.9821; observed 278.9817.

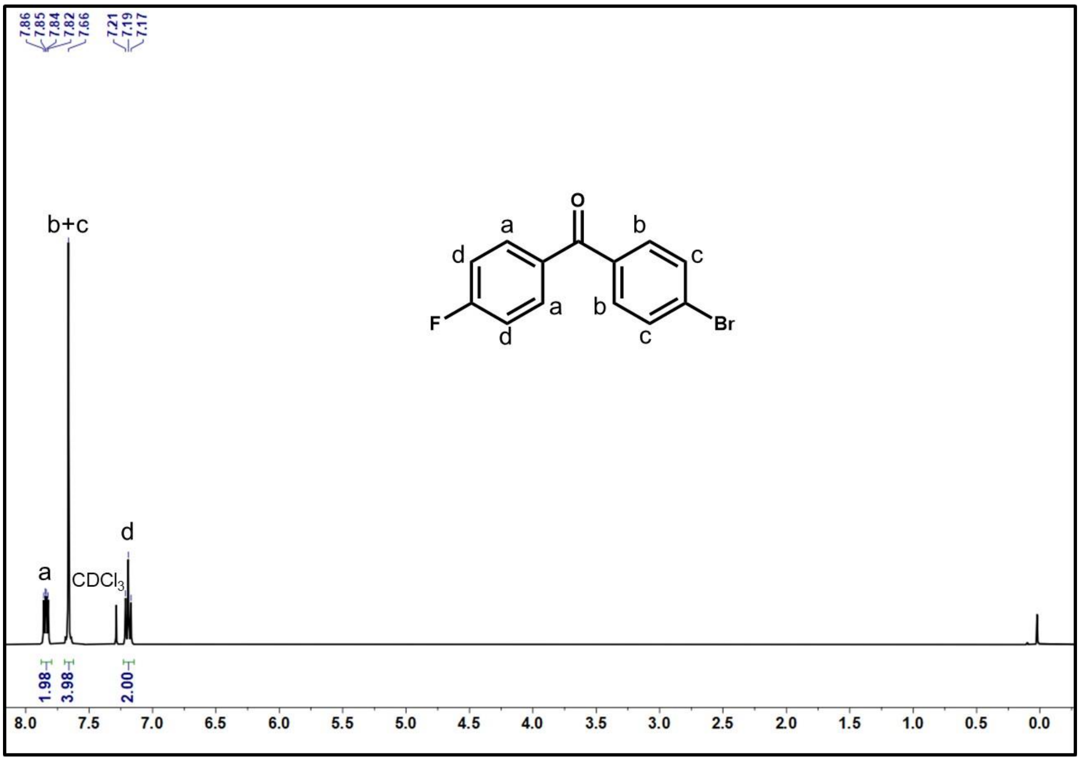

Figure S1. The ^{1}HNMR of 4-fluoro-4'-bromo-dibenzophenone.

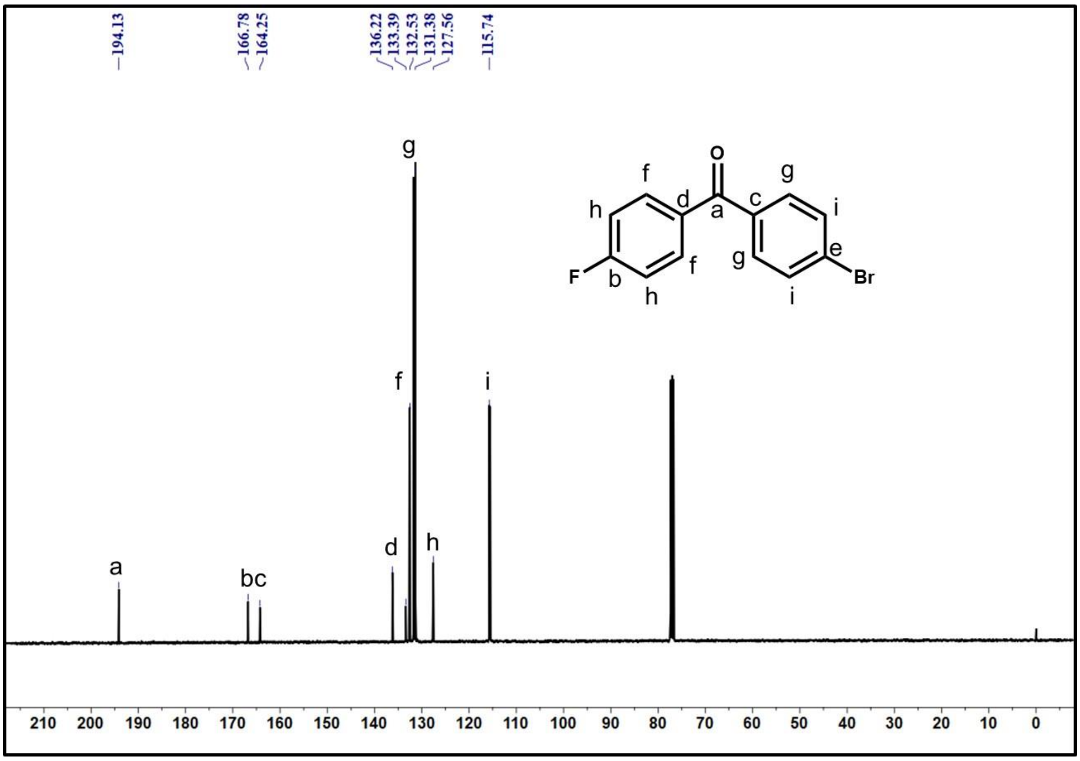

Figure S2. The ^{13}CNMR of 4-fluoro-4'-bromo-dibenzophenone.

b. Synthesis of **BD-*Br***

9, 9-Dimethyl acridine (1151.1 mg, 5.5 mmol) was dissolved in 50 mL DMF and stirred in argon atmosphere at 0 ºC. Then the anhydrous *tert*-butanol potassium (841.6 mg, 7.5 mmol) was added to the reaction system in batches and stirred for 5 h at 0 ºC. After that, **F-BP-Br** (1395.6 mg, 5 mmol) was added to the reaction system slowly, and then the mixture was stirred for another 12 hours after the reaction system returned to room temperature naturally. Then the system temperature was raised to 60 ºC and continued to be stirred for 12 hours. When the reaction system was returned to room temperature naturally, the reactants were poured into a solution of dilute hydrochloric acid to quench the reaction. After washing for three times by water, the organic phase was extracted with dichloromethane. Then the organic phase was dried with anhydrous magnesium sulfate, filtered and concentrate to obtain the crude product. The crude product was further purified by column chromatography on silica gel using petroleum ether/dichloromethane (*v*/*v* = 5/1) as eluent to obtain the compound of **BD-*Br*** as a yellow-green powder (1.69 g, 72% yield).

^{1}H NMR (400 MHz, $CDCl_3$) δ 8.04 (d, *J* = 8.1 Hz, 2H), 7.78 (d, *J* = 8.2 Hz, 2H), 7.69 (d, *J* = 8.2 Hz, 2H), 7.53-7.44 (m, 4H), 7.05-6.91 (m, 4H), 6.33 (d, *J* = 9.4 Hz, 2H), 1.69 (s, 6H).

^{13}C NMR (101 MHz, $CDCl_3$) δ 194.74, 145.75, 140.44, 136.48, 136.08, 132.59, 131.83, 131.57, 130.88, 130.83, 127.85, 126.43, 125.39, 121.22, 114.45, 36.12, 31.07.

LC-MS m/z [M+H] calculated for C13H8FBrO+H 469.0963; observed 469.0960.

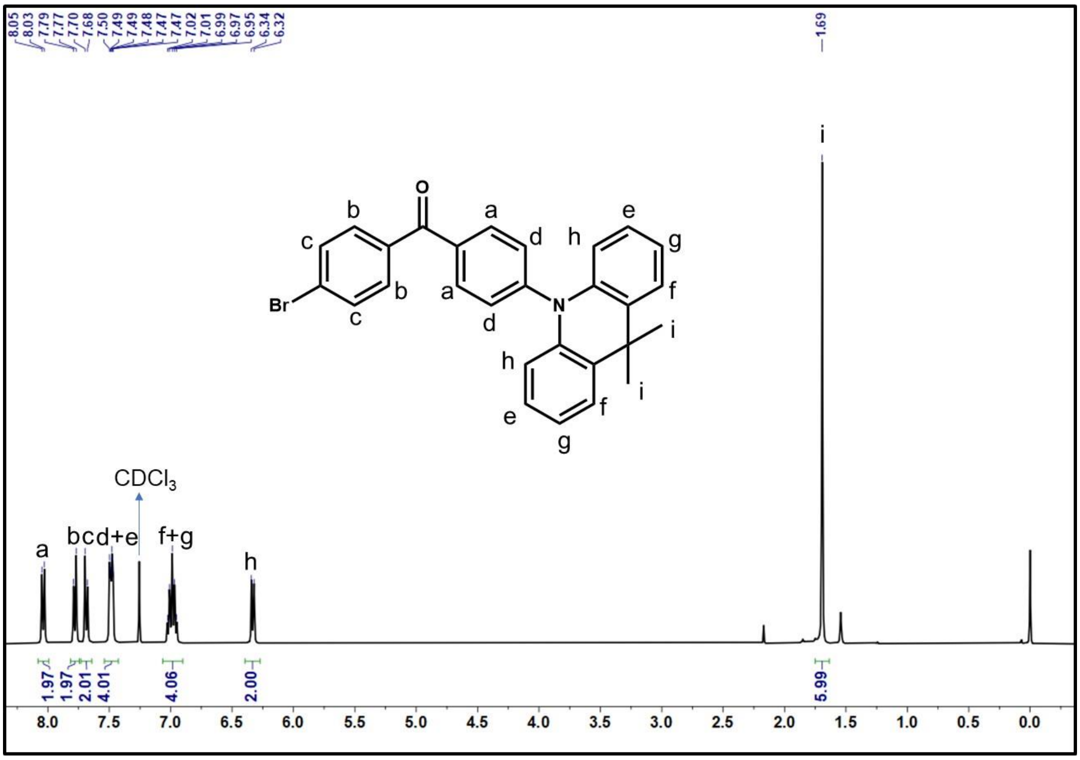

Figure S3. The ^{1}HNMR of 4-bromo-4'-(9, 9-dimethyl acridine)-dibenzophenone.

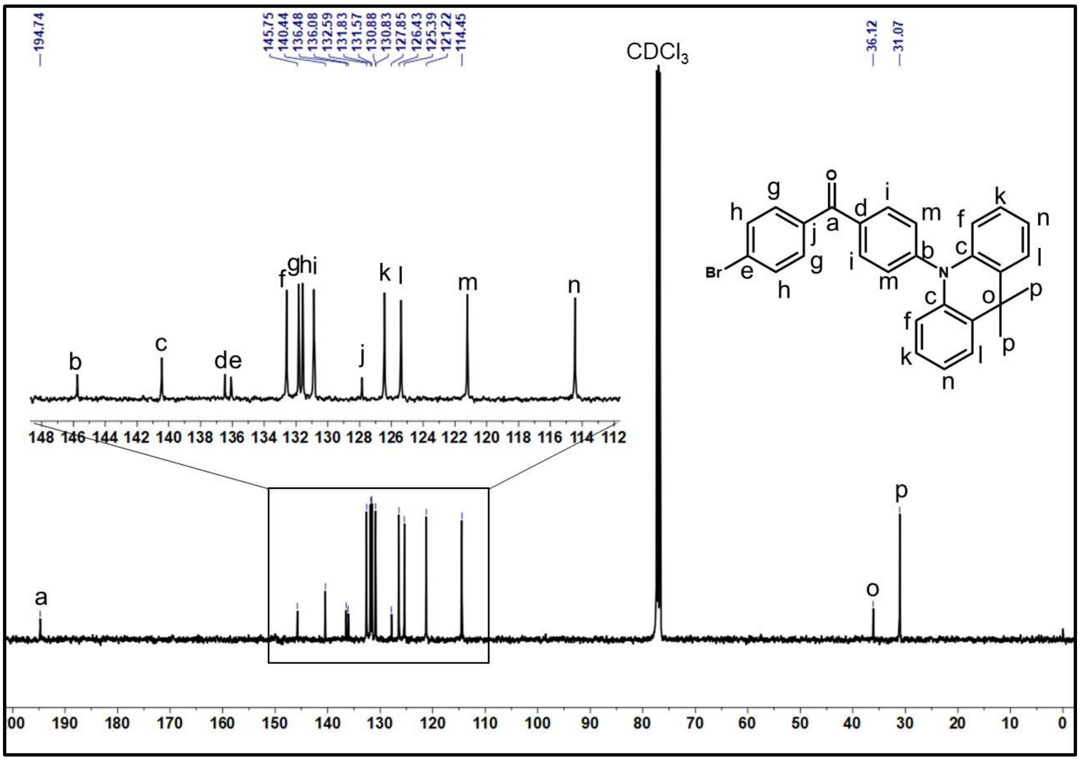

Figure S4. The ^{13}CNMR of 4-bromo-4'-(9, 9-dimethyl acridine)-dibenzophenone.

S2. Calculation and photophysical properties

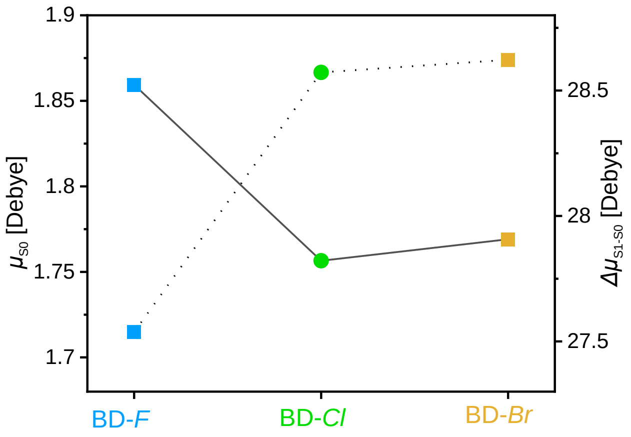

Figure S5. The molecular dipole moment (μ_{s0}) and transition dipole moment ($\Delta\mu_{s1\text{-}s0}$) for BD-*F*, BD-*Cl* and BD-*Br*.

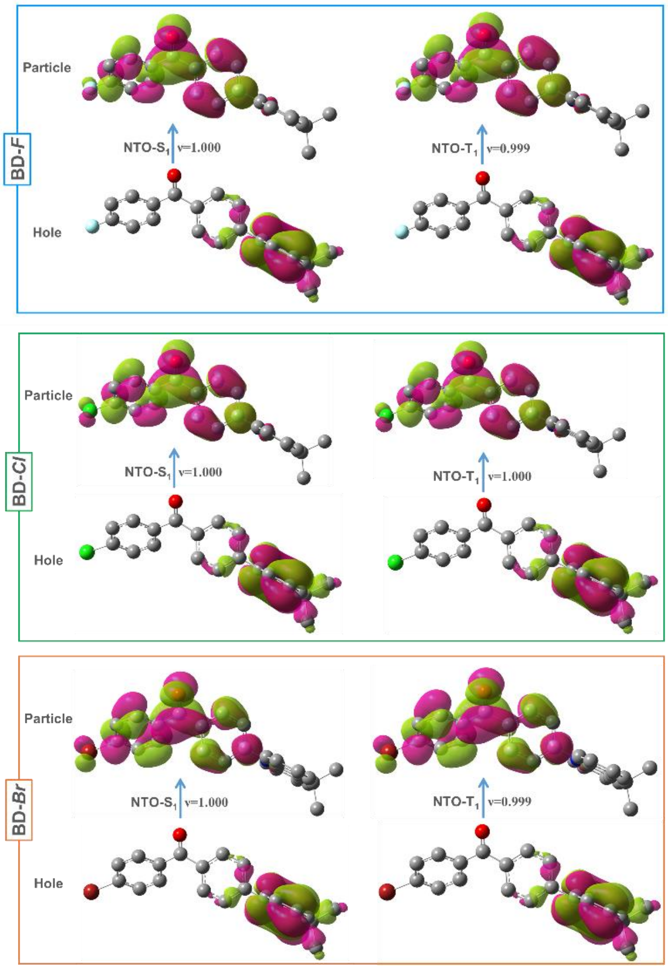

Figure S6. Natural transition orbital (NTO) analyses of the lowest singlet (S_1) and triplet (T_1) excited states of the halogenated emitters.

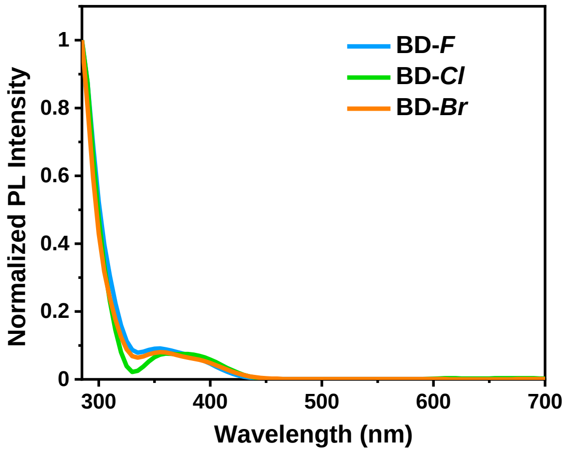

Figure S7. The ultraviolet-visible absorption spectra of emitters diluted in toluene.

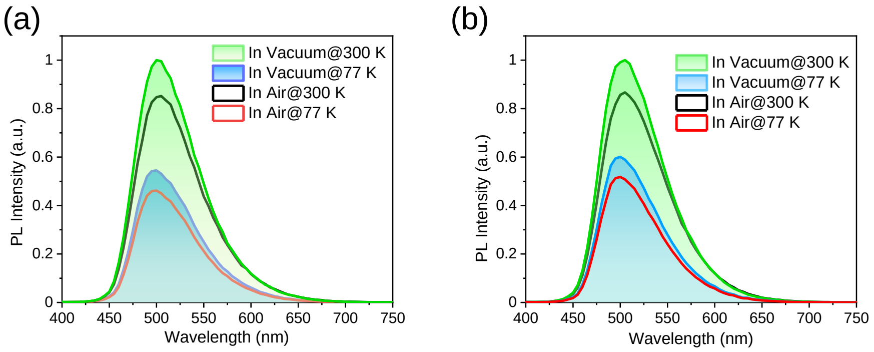

Figure S8. The steady-state PL spectra of blended films for (a) BD-*Cl* and (b) BD-*Br* in air or vacuum at room temperature and 77 K.

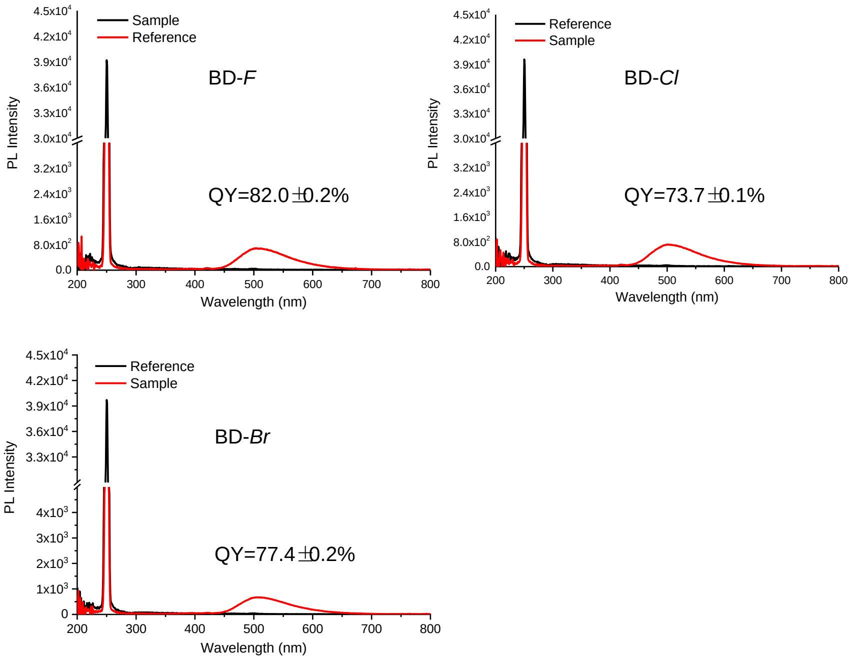

Figure S9. The photoluminescence quantum yield detected in the blended film in air.

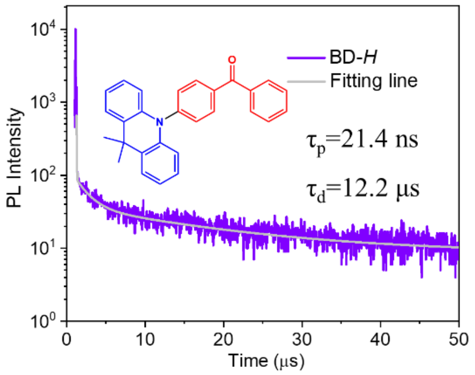

Figure S10. The transient PL decay spectra of the blended film for BD-*H* in vacuum. The molecular structure and lifetime of DF and PF component are also shown above.

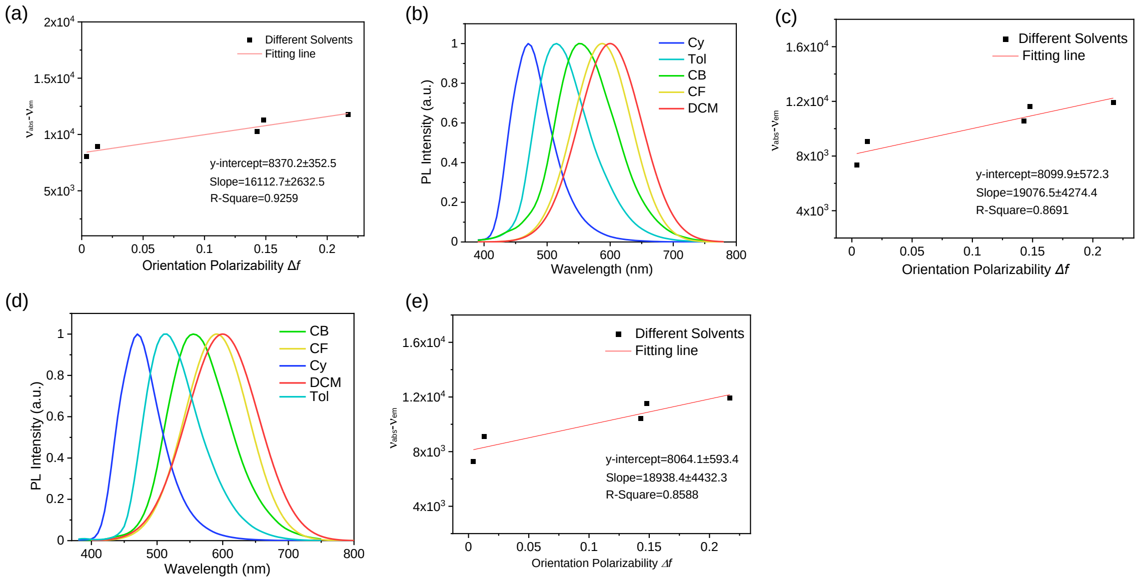

Figure S11. Emission spectra of BD-F diluted in different solvents at room temperature and the corresponding Lippert-Mataga plot for (a) BD-*F*, (b)(c) BD-*Cl* and (d)(e) BD-Br.

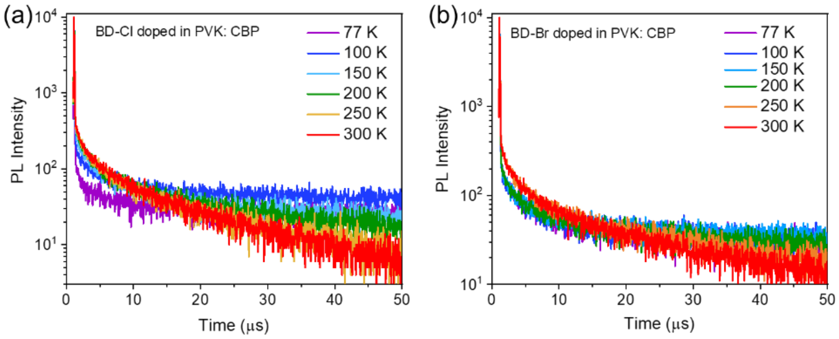

Figure S12. The temperature-dependent transient PL decay spectra of the blended film for (a) BD-*Cl* and (b) BD-*Br*.

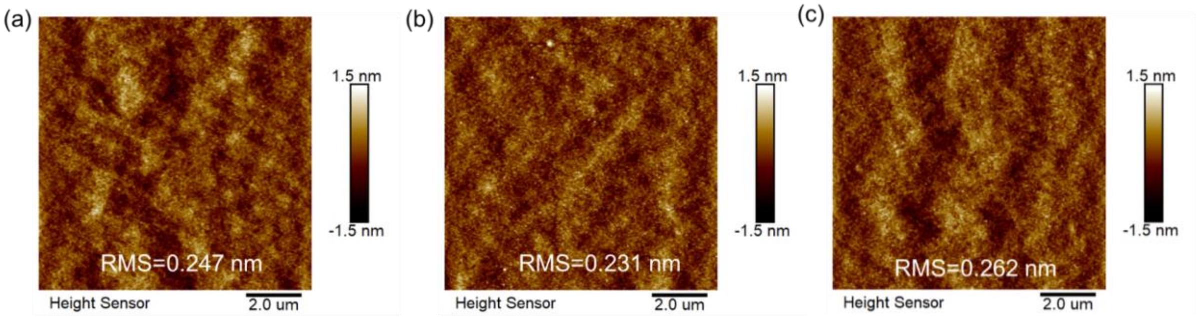

Figure S13. The morphology of the blended films detected by atomic force microscopy for (a) BD-*F*, (b) BD-*Cl* and (c) BD-*Br*. The root mean-square (RMS) surface roughness of the whole scanning area are shown in the images.

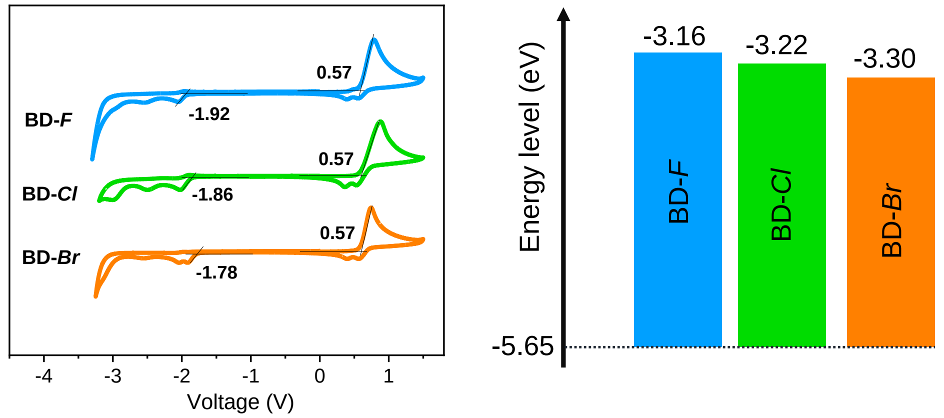

Figure S14. The Cyclic voltammetry of halogenated emitters and the corresponding energy levels of HOMO and LUMO.

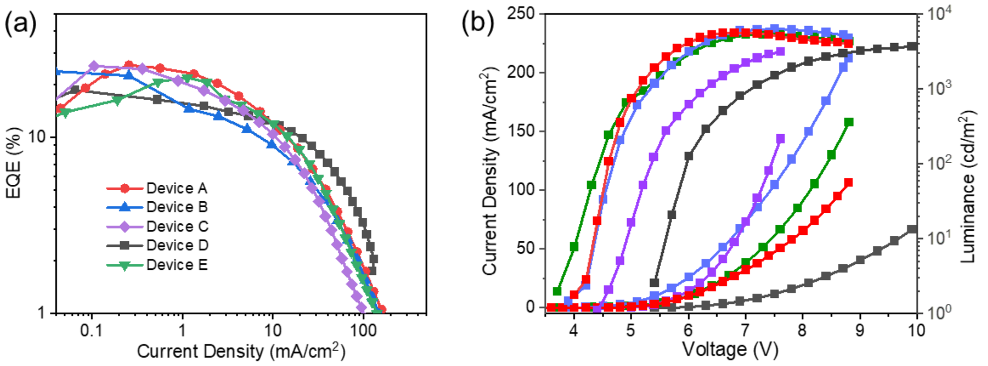

Figure S15. (a) The curves of external quantum efficiency (EQE) versus current density. (b) The current density-voltage-luminance curves. ITO/PSS:PEDOT/A. 10wt% BD-*F*: 20 wt% PVK: 70 wt% CBP; B. 30wt% BD-*F*: 20 wt% PVK: 50 wt% CBP; C. 20wt% BD-*F*: 30 wt% PVK: 50 wt% CBP; D. 20wt% BD-*F*: 20 wt% PVK: 60 wt% 2,2′-(1,3-Phenylene)-bis[5-(4-tert-butylphenyl)-1,3,4-oxadiazole] (OXD-7); E. 20wt% BD-*F*: 20 wt% PVK: 60 wt% 9-(3-(9H-carbazol-9-yl)phenyl)-9H-carbazole-3-carbonitrile (mCPCN) /DPEPO/TmPyPB/LiF/Al.

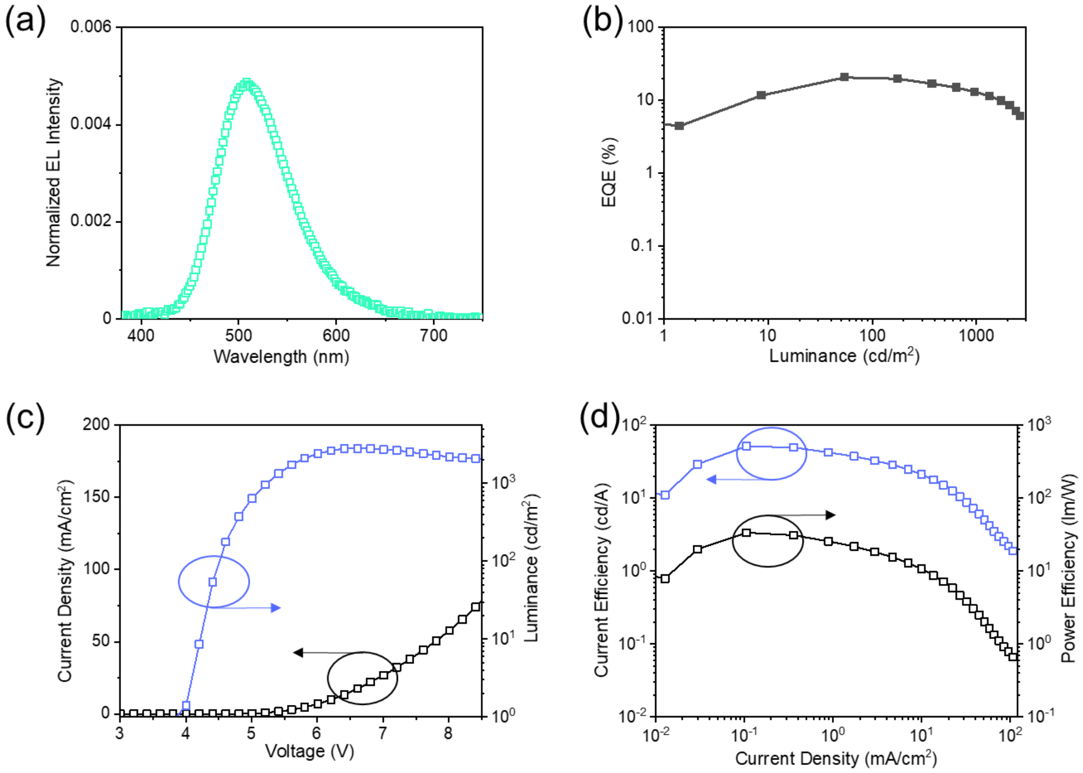

Figure S16. The solution-processable OLEDs performance of BD-*H* based device (Device F). (a) The electroluminescence spectra detected at 5 V. (b) The curves of external quantum efficiency (EQE) versus luminance. (c) Current density-voltage-luminance curves. (d) The curves of power efficiency and current efficiency versus current density.

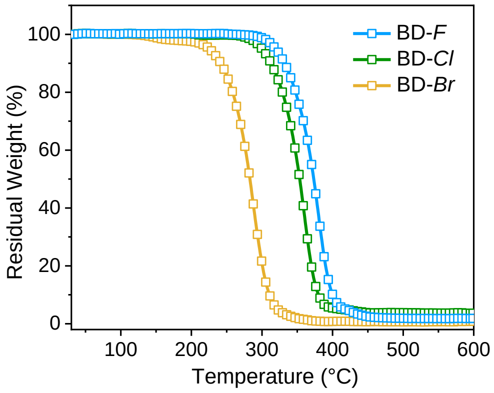

Figure S17. TGA curves of these emitters at a heating rate of 10 °C min^{-1} under N_2.

S 3. Single crystal X-ray data

(1) BD-*F*, CCDC number: 2034535

**Space-group**	P 1 (1)-triclinic

Cell	a=15.8563(12) Å b=8.0739(6) Å c=16.4160(12) Å α=90.0000 β=91.329(7) γ=90.0000 V=2101.05(30) Å^3 Z=4

Atomic parameters							
Atom	Wyck.	Site	x/a	y/b	z/c	U[Å^2]	Flag
N1	1a	1	0.6499(1)	0.4955(2)	0.0841(1)		
O1	1a	1	0.7036(1)	1.0316(2)	-0.1923(1)		
C7	1a	1	0.7462(1)	1.0182(3)	-0.1294(1)		
F1	1a	1	1.0004(1)	1.4911(2)	-0.0813(1)		
C11	1a	1	0.6798(1)	0.6259(3)	0.0317(1)		
C20	1a	1	0.5422(1)	0.3954(3)	0.1748(1)		
C19	1a	1	0.5812(1)	0.2234(3)	0.1664(1)		
C13	1a	1	0.7076(1)	0.3762(3)	0.1150(1)		
C27	1a	1	0.5500(1)	0.1513(3)	0.0838(2)		
H27A	1a	1	0.48990	0.13610	0.08460	0.0400	calc
H27B	1a	1	0.57680	0.04650	0.07480	0.0400	calc
H27C	1a	1	0.56370	0.22640	0.04080	0.0400	calc
C25	1a	1	0.5744(1)	0.5238(3)	0.1269(1)		
C10	1a	1	0.7196(1)	0.7653(3)	0.0637(1)		
H10	1a	1	0.72980	0.77400	0.11960	0.0300	calc
C21	1a	1	0.4744(1)	0.4302(3)	0.2246(1)		
H21	1a	1	0.45450	0.34830	0.25910	0.0290	calc
C22	1a	1	0.4359(1)	0.5849(3)	0.2237(2)		
H22	1a	1	0.39170	0.60700	0.25830	0.0320	calc
C6	1a	1	0.8139(1)	1.1420(3)	-0.1111(1)		
C17	1a	1	0.7342(1)	0.1361(3)	0.1988(1)		
H17	1a	1	0.71470	0.05260	0.23250	0.0310	calc
C9	1a	1	0.7442(1)	0.8916(3)	0.0123(1)		
H9	1a	1	0.77070	0.98520	0.03380	0.0300	calc
C23	1a	1	0.4638(1)	0.7059(3)	0.1711(2)		
H23	1a	1	0.43610	0.80730	0.16780	0.0320	calc
C18	1a	1	0.6767(1)	0.2448(3)	0.1624(1)		
C24	1a	1	0.5332(1)	0.6758(3)	0.1231(2)		
H24	1a	1	0.55220	0.75790	0.08830	0.0290	calc
C4	1a	1	0.8655(2)	1.4201(3)	-0.1307(2)		
H4	1a	1	0.85810	1.52700	-0.15070	0.0490	calc
C16	1a	1	0.8201(2)	0.1494(3)	0.1857(2)		
H16	1a	1	0.85780	0.07630	0.21090	0.0350	calc
C8BA	1a	1	0.6657(1)	0.6107(3)	-0.0515(1)		
H8B	1a	1	0.63990	0.51630	-0.07280	0.0300	calc
C8	1a	1	0.7293(1)	0.8790(3)	-0.0720(1)		
C12	1a	1	0.6905(1)	0.7375(3)	-0.1027(2)		
H12	1a	1	0.68080	0.72760	-0.15850	0.0310	calc
C14	1a	1	0.7936(1)	0.3860(3)	0.0998(1)		
H14	1a	1	0.81380	0.46900	0.06620	0.0300	calc
C5	1a	1	0.8036(2)	1.3028(3)	-0.1410(2)		
H5	1a	1	0.75360	1.33100	-0.16840	0.0400	calc
C15	1a	1	0.8491(1)	0.2727(3)	0.1346(2)		
H15	1a	1	0.90630	0.27920	0.12370	0.0330	calc
C26	1a	1	0.5558(2)	0.1055(3)	0.2341(2)		
H26A	1a	1	0.57550	0.14820	0.28570	0.0450	calc
H26B	1a	1	0.58050	-0.00140	0.22510	0.0450	calc
H26C	1a	1	0.49550	0.09530	0.23420	0.0450	calc
C1	1a	1	0.8887(2)	1.1019(3)	-0.0705(2)		
H1	1a	1	0.89640	0.99580	-0.04960	0.0430	calc
C3	1a	1	0.9378(2)	1.3760(4)	-0.0905(2)		
C2	1a	1	0.9527(2)	1.2203(4)	-0.0604(2)		
H2	1a	1	1.00350	1.19400	-0.03420	0.0540	calc

(2) BD-*Cl*, CCDC number: 2034536

Space-group	P 1 21/c 1 (14) - monoclinic
Cell	a=12.1917(7) Å b=8.0116(6) Å c=22.3369(15) Å β=94.300(6) V=2175.62(20) Å^3 Z=4

Atomic parameters

Atom	Wyck.	Site	S.O.F.	x/a	y/b	z/c	U[Å^2]	Flag
Cl1	4e	1	0.918(3)	0.0574(1)	0.0925(2)	0.29820		
O1	4e	1		-0.0974(1)	-0.3847(2)	0.5216(1)		
N1	4e	1		-0.4040(2)	-0.9282(3)	0.3825(1)		
C7	4e	1		-0.1135(2)	-0.4043(3)	0.4672(1)		
C21	4e	1		-0.5884(2)	-1.0414(3)	0.3833(1)		
C8	4e	1		-0.1850(2)	-0.5426(3)	0.4422(1)		
C22	4e	1		-0.6985(2)	-1.0164(3)	0.3931(1)		
H22	4e	1		-0.74830	-1.10260	0.38460	0.0280	calc
C11	4e	1		-0.3282(2)	-0.7978(3)	0.4012(1)		
C10	4e	1		-0.3088(2)	-0.6648(3)	0.3641(1)		
H10	4e	1		-0.34360	-0.65980	0.32560	0.0290	calc
C19	4e	1		-0.4497(2)	-1.1771(3)	0.3241(1)		
C24	4e	1		-0.6623(2)	-0.7388(3)	0.4280(1)		
H24	4e	1		-0.68650	-0.63900	0.44370	0.0300	calc
C16	4e	1		-0.2595(2)	-1.1385(4)	0.2628(1)		
H16	4e	1		-0.19470	-1.12860	0.24360	0.0350	calc
C26	4e	1		-0.5153(2)	-0.9082(3)	0.3950(1)		
C12	4e	1		-0.2774(2)	-0.8038(3)	0.4591(1)		
H12	4e	1		-0.29080	-0.89300	0.48420	0.0280	calc
C14	4e	1		-0.3772(2)	-1.0434(3)	0.3379(1)		
C15	4e	1		-0.2811(2)	-1.0281(3)	0.3080(1)		
H15	4e	1		-0.23140	-0.94310	0.31850	0.0300	calc
C17	4e	1		-0.3335(2)	-1.2634(4)	0.2461(1)		
H17	4e	1		-0.32060	-1.33440	0.21440	0.0350	calc
C25	4e	1		-0.5527(2)	-0.7583(3)	0.4178(1)		
H25	4e	1		-0.50360	-0.67100	0.42610	0.0260	calc
C13	4e	1		-0.2069(2)	-0.6767(3)	0.4792(1)		
H13	4e	1		-0.17370	-0.68100	0.51810	0.0300	calc
C9	4e	1		-0.2370(2)	-0.5379(3)	0.3842(1)		
H9	4e	1		-0.22360	-0.44930	0.35890	0.0280	calc
C20	4e	1		-0.5414(2)	-1.2094(3)	0.3656(1)		
C18	4e	1		-0.4270(2)	-1.2819(4)	0.2768(1)		
H18	4e	1		-0.47630	-1.36700	0.26560	0.0360	calc
C23	4e	1		-0.7360(2)	-0.8670(4)	0.4151(1)		
H23	4e	1		-0.81010	-0.85310	0.42110	0.0310	calc
C3	4e	1		0.0149(3)	-0.0509(5)	0.3496(2)		
C5	4e	1		-0.0706(2)	-0.1154(4)	0.4383(1)		
H5	4e	1		-0.10060	-0.08100	0.47330	0.0420	calc
C27	4e	1		-0.6306(2)	-1.3271(3)	0.3373(1)		
H27A	4e	1		-0.68480	-1.34710	0.36550	0.0500	calc
H27B	4e	1		-0.59760	-1.43100	0.32700	0.0500	calc
H27C	4e	1		-0.66510	-1.27660	0.30170	0.0500	calc
C28	4e	1		-0.4885(2)	-1.2913(4)	0.4235(1)		
H28A	4e	1		-0.43400	-1.21760	0.44210	0.0450	calc
H28B	4e	1		-0.45430	-1.39470	0.41350	0.0450	calc
H28C	4e	1		-0.54420	-1.31250	0.45070	0.0450	calc
C6	4e	1		-0.0660(2)	-0.2846(4)	0.4253(1)		
C4	4e	1		-0.0318(2)	0.0027(4)	0.4004(2)		
H4	4e	1		-0.03700	0.11590	0.40890	0.0560	calc
C1	4e	1		-0.0163(2)	-0.3358(4)	0.3747(1)		
H1	4e	1		-0.01110	-0.44900	0.36610	0.0460	calc
C2	4e	1		0.0260(2)	-0.2185(5)	0.3366(1)		
H2	4e	1		0.06090	-0.25220	0.30300	0.0590	calc
Cl1A	4e	1	0.082(3)	0.0237(12)	-0.5214(18)	0.3482(6)		

(3) BD-*Br*, CCDC number: 2034537

**Space-group**	P 1 21/c 1 (14) - monoclinic
**Cell**	a=13.3742(6) Å b=20.6493(8) Å c=8.1034(3) Å β=91.848(2)<br>V=2236.73(20) Å^3 Z=4

Atom	Wyck.	Site	x/a	y/b	z/c	U[Å^2]	Flag

Br1	4e	1	0.4083(1)	0.67460	0.9812(1)		
O1	4e	1	0.8502(4)	0.7072(2)	0.5348(6)		
N1	4e	1	0.8039(4)	0.4800(3)	0.0031(7)		
C1	4e	1	0.8342(7)	0.3379(4)	-0.3793(11)		
H18	4e	1	0.90240	0.32340	-0.40010	0.0920	calc
H17	4e	1	0.80040	0.34990	-0.48410	0.0920	calc
H1	4e	1	0.79720	0.30290	-0.32730	0.0920	calc
C2	4e	1	0.8381(6)	0.3971(4)	-0.2639(8)		
C3	4e	1	0.8794(5)	0.3807(3)	-0.0918(8)		
C4	4e	1	0.8666(5)	0.4256(3)	0.0336(8)		
C5	4e	1	0.7932(5)	0.5280(3)	0.1318(8)		
C6	4e	1	0.7406(5)	0.5154(3)	0.2688(8)		
H19	4e	1	0.70900	0.47450	0.28010	0.0500	calc
C7	4e	1	0.7325(5)	0.5615(3)	0.3927(8)		
H3	4e	1	0.69470	0.55260	0.48740	0.0490	calc
C8	4e	1	0.7809(5)	0.6214(3)	0.3758(8)		
C9	4e	1	0.7792(5)	0.6696(3)	0.5119(9)		
C10	4e	1	0.6907(6)	0.6722(3)	0.6216(10)		
C11	4e	1	0.7058(7)	0.6831(3)	0.7893(9)		
H2	4e	1	0.77190	0.68960	0.83300	0.0580	calc
C12	4e	1	0.6273(7)	0.6847(4)	0.8928(10)		
H22	4e	1	0.63870	0.69050	1.00820	0.0680	calc
C13	4e	1	0.5297(7)	0.6778(3)	0.8274(12)		
C14	4e	1	0.8403(5)	0.5869(3)	0.1120(8)		
H4	4e	1	0.87740	0.59530	0.01630	0.0490	calc
C15	4e	1	0.8325(5)	0.6336(3)	0.2344(9)		
H5	4e	1	0.86310	0.67460	0.22040	0.0520	calc
C16	4e	1	0.9348(6)	0.3251(3)	-0.0546(11)		
H6	4e	1	0.94070	0.29260	-0.13670	0.0590	calc
C17	4e	1	0.9813(5)	0.3160(3)	0.0977(9)		
H9	4e	1	1.01880	0.27770	0.12000	0.0530	calc
C18	4e	1	0.9731(5)	0.3621(4)	0.2159(10)		
H7	4e	1	1.00690	0.35640	0.31990	0.0580	calc
C19	4e	1	0.9164(5)	0.4173(4)	0.1874(8)		
H8	4e	1	0.91120	0.44930	0.27090	0.0530	calc
C20	4e	1	0.7376(5)	0.4299(3)	-0.2458(8)		
C21	4e	1	0.6567(6)	0.4210(4)	-0.3568(9)		
H10	4e	1	0.66240	0.39050	-0.44380	0.0590	calc
C22	4e	1	0.5684(5)	0.4555(4)	-0.3435(10)		
H13	4e	1	0.51350	0.44760	-0.41820	0.0620	calc
C23	4e	1	0.5610(5)	0.5010(4)	-0.2212(9)		
H11	4e	1	0.50200	0.52630	-0.21550	0.0590	calc
C24	4e	1	0.6374(5)	0.5104(3)	-0.1073(9)		
H12	4e	1	0.63070	0.54170	-0.02230	0.0550	calc
C25	4e	1	0.7258(5)	0.4738(3)	-0.1161(8)		
C26	4e	1	0.9094(6)	0.4485(4)	-0.3374(9)		
H15	4e	1	0.91630	0.48520	-0.26120	0.0780	calc
H14	4e	1	0.88150	0.46360	-0.44380	0.0780	calc
H16	4e	1	0.97520	0.42900	-0.35330	0.0780	calc
C27	4e	1	0.5948(5)	0.6658(3)	0.5564(10)		
H20	4e	1	0.58410	0.65900	0.44120	0.0550	calc
C28	4e	1	0.5142(6)	0.6693(3)	0.6586(11)		
H21	4e	1	0.44800	0.66590	0.61320	0.0640	calc

Table S1. Elemental analyses data for the emitters[a]

Emitter	C [wt%]	N [wt%]	H [wt%]

BD-*H*	85.94[b]/86.26[c]	3.51/3.59	6.10/5.95
BD-*F*	82.39/82.53	3.29/3.44	5.57/5.44
BD-*Cl*	79.30/79.25	3.28/3.25	5.26/5.23
BD-*Br*	71.77/71.73	2.96/2.98	4.81/4.73

[a] Performed under varioEL cube V2.0.1 (755b431)2010-12-16,CHNS Mode,Ser.NO:19106057 Elementar Analysensysteme GmbH; [b] the experimental data. [c] the calculated data.

Table S2. Summary of quantum chemical calculation results.

Emitter	Transition	S_r value	CT ratio [%]	Nature of excited states	$\langle S_1 \| \hat{H}_{SOC} \| T_1 \rangle$ [cm^{-1}]
BP-*F*	S_1-S_0	0.1262	87.4	CT	0
	T_1-S_0	0.1385	86.2	CT	
BP-*Cl*	S_1-S_0	0.1210	87.9	CT	0
	T_1-S_0	0.1343	86.6	CT	
BP-*Br*	S_1-S_0	0.1210	87.9	CT	0.01
	T_1-S_0	0.1342	86.6	CT	

Table S3. The kinetic decay lifetime of triplet excitons detected at 725 nm.

Emitter	A_1 [10^{-3}]	τ_1 (ns)	A_2 [10^{-3}]	τ_2 (ns)	τ_{ave} (ns)
BD-*F*	4.74	3.04	3.22	0.23	2.90
BD-*Cl*	2.57	0.21	4.91	2.07	1.98
BD-*Br*	0.98	0.065	3.85	1.93	1.92

Using expdecay2 to fitting the decay curves, $\tau_{ave} = \frac{A_1\tau_1^2+A_2\tau_2^2}{A_1\tau_1+A_2\tau_2} = \frac{\sum A_i\tau_i^2}{\sum A_i\tau_i}$.

Table S4. The quantum efficiency (Φ_{PL}) and device performance of solution-processable OLEDs

Emitter	Φ_{PL} [%]	V_{on} [V]	L_{max} [cd/m^2]	CE_{max} [cd/A]	CE_{max} [lm/W]	EQE [%]
Device A. 10 wt% BD-*F*: 20 wt% PVK: 70 wt% CBP	77.7	3.7	5,632	68.8	45.4	25.7
Device B. 30 wt% BD-*F*: 20 wt% PVK: 50 wt% CBP	70.3	3.7	6,350	60.7	42.0	23.7
Device C. 20 wt% BD-*F*: 30 wt% PVK: 50 wt% CBP	73.5	4.3	3,453	65.4	44.3	25.4
Device D. 20 wt% BD-*F*: 20 wt% PVK: 60 wt% OXD-7	62.0	5.3	3,687	48.6	28.6	18.5
Device E. 20 wt% BD-*F*: 20 wt% PVK: 60 wt% mCPCN	66.5	3.5	5,386	59.3	35.8	21.8
Device F. 20 wt% BD-*H*: 20 wt% PVK: 60 wt% CBP	64.3	4.0	2,816	51.4	33.6	20.6

Reference

[1] Y. Liu, Y. Wang, C. Li, Z. Ren, D. Ma, S. Yan, *Macromolecules* **2018**, 51, 4615.

[2] H. Sun, C. Zhong, J. L. Brédas, *J. Chem. Theory Comput*. **2015**, 11, 3851.

[3] T. Lu, F. Chen, *J. Comput. Chem.* **2012**, 33, 580.

[4] T. Lu, F. Chen, *Acta Chim. Sinica* **2011**, 69, 2393.

[5] S. Chen, N. Ullah, R. Zhang, *J. Phys. Chem. Lett.* **2018**, 9, 4857.

[6] Y. C. Liu, L. Hua, S. K. Yan, Z. J. Ren, *Nano Energy* **2020**, 73, 104800.
